# The Regulation of Intestinal Inflammation and Cancer Development by Type 2 Immune Responses

**DOI:** 10.3390/ijms21249772

**Published:** 2020-12-21

**Authors:** Reyes Gamez-Belmonte, Lena Erkert, Stefan Wirtz, Christoph Becker

**Affiliations:** Department of Medicine 1, University of Erlangen-Nuremberg, 91052 Erlangen, Germany; MariadelosReyes.GamezBelmonte@uk-erlangen.de (R.G.-B.); lena.erkert@uk-erlangen.de (L.E.); stefan.wirtz@uk-erlangen.de (S.W.)

**Keywords:** intestinal inflammation, colon cancer, type 2 immunity

## Abstract

The gut is among the most complex organs of the human body. It has to exert several functions including food and water absorption while setting up an efficient barrier to the outside world. Dysfunction of the gut can be life-threatening. Diseases of the gastrointestinal tract such as inflammatory bowel disease, infections, or colorectal cancer, therefore, pose substantial challenges to clinical care. The intestinal epithelium plays an important role in intestinal disease development. It not only establishes an important barrier against the gut lumen but also constantly signals information about the gut lumen and its composition to immune cells in the bowel wall. Such signaling across the epithelial barrier also occurs in the other direction. Intestinal epithelial cells respond to cytokines and other mediators of immune cells in the lamina propria and shape the microbial community within the gut by producing various antimicrobial peptides. Thus, the epithelium can be considered as an interpreter between the microbiota and the mucosal immune system, safeguarding and moderating communication to the benefit of the host. Type 2 immune responses play important roles in immune-epithelial communication. They contribute to gut tissue homeostasis and protect the host against infections with helminths. However, they are also involved in pathogenic pathways in inflammatory bowel disease and colorectal cancer. The current review provides an overview of current concepts regarding type 2 immune responses in intestinal physiology and pathophysiology.

## 1. Introduction

With a surface of more than 30 m^2^, the primary functions of the gut are digestion and selective absorption of nutrients. The gut surface comprises a monolayer of intestinal epithelial cells covered by mucus, a selective and permeable physical barrier that prevents the translocation of microbial antigens and food allergens while allowing the absorption of water and nutrients [1]. The second component of host defense is the mucosal immune system, with immune cell populations, regulating and executing both adaptive and innate immune responses, making the gut the largest immune organ in our body. The delicate task of the gut immune system is to trigger inflammatory responses against pathogenic microorganisms while establishing immunological tolerance against harmless food allergens and commensal bacteria [2]. Finally, the gastrointestinal tract harbors a microbiome with a population of about 100 trillion cells. Its composition plays an important role in establishing and maintaining gut tissue and immune homeostasis, prerequisites of human health [3].

Recent scientific achievements have positioned type 2 immune responses into the spotlight regarding their role in intestinal homeostasis but also in chronic inflammatory disorders. Type 2 immunity includes cells and molecules traditionally associated with the response to allergens and helminth infections. Nevertheless, new evidence has demonstrated the decisive role of this type of immune response in other key tasks such as immunosurveillance and wound healing. In the classic view of type 2 immune responses, allergens or parasitic infections trigger the differentiation of CD4^+^ T cells toward a Th2 phenotype. Th2 cells are characterized by the release of cytokines such as interleukin (IL)-4, IL-5, IL-9, and IL-13 [4]. However, it has become evident that type 2 immune responses exhibit a greater complexity. Alternatively activated macrophages, eosinophils, basophils, and mast cells are among the effector cells also associated with type 2 immunity. More recently, the discovery of innate lymphoid cells (ILC) has shed some light on the development of different immune responses in mucosa-associated tissues, such as the gastrointestinal tract. ILC2s are, together with Th2 cells, an important source of type 2 cytokines in the gut. ILC2 can sense various epithelial-derived cytokines and have been demonstrated to constitute an early effector in type 2 immunity [5,6]. About ten years ago, a new population of CD4^+^ T cells, characterized by the production of IL-9, was described and denoted as Th9 cells. IL-9 is a pleiotropic type 2 cytokine involved in anti-parasitic and allergic reactions. Th9 cells are currently under extensive investigation and recently, Th2 cells were proposed as an intermediate state in the differentiation of Th9 cells [7].

Type 2 mediators can contribute to gut homeostasis, e.g., some type 2 cytokines have been associated with mucus production and intestinal stem cell renewal. However, when dysregulated, type 2 immunity contributes to the pathogenesis of gut-related diseases, including inflammatory bowel disease (IBD) and colorectal cancer (CRC). In the former, IL-4 and IL-13 have been shown to modulate intestinal inflammation in experimental models, which led to the development of anti-IL-13 agents that were evaluated in clinical trials. However, considering type 2 effector cells, type cell dependent-positive and negative effects have been described. Nevertheless, the promising results obtained in a helminth-based therapy in IBD suggest a beneficial role of the classic type 2 immune response in this pathogenic condition. On the contrary, in the case of CRC, increasing evidence points to a deleterious role of type 2 immunity in tumor development.

Given these new findings, a better understanding of type 2 immunity in the gut holds promise for future therapeutic approaches. In this review, we will focus on the role of type 2 immunity in the gut in health and disease, with particular attention to the involvement of type 2 immunity in IBD and CRC.

## 2. The Role of Type 2 Immunity in the Gut: From Homeostasis to Disease

Type 2 immunity activation can exhibit either pathogenic or host-protective activity, e.g., during allergic responses or helminthic infection, respectively. However, there is evidence indicating that type 2 associated cytokines and cells can also have important functions under steady-state conditions [8]. In fact, non-immune cells in the intestine express the receptor chains for the two key type 2 cytokines, that is, IL-4 and IL-13. Regarding the role of these type 2 cytokines, recent evidence suggests that IL-13 signaling could be involved in intestinal stem cell (ISC) self-renewal and intestinal homeostasis. Accordingly, using conditional deletion of *Il13ra1* in Lgr5-GFP^+^ ISCs, Zhu et al. elegantly described structural changes, such as a reduced length of crypts and villi in these mice. Since this effect was not observed in IL4^-/-^ mice, they proposed a pathway in which ILC2-derived IL-13 promotes intestinal stem cell renewal via STAT6 and Foxp1dependent activation of the β-catenin pathway in crypt ISCs [9]. Moreover, type 2 immunity has been demonstrated to play a relevant role in the modulation of mucus secretion from goblet cells. As such, ILC2 constitutively express IL-5, a crucial cytokine in the determination of steady-state eosinophil numbers. The importance of eosinophils in regulating mucus secretion is highlighted by the observation that their absence leads to a reduction in the number of mucus-secreting goblet cells [10]. Furthermore, intestinal tuft cells maintain ILC2 homeostasis via the release of IL-25 [11]. Finally, some type 2 immune cells, such as mast cells or macrophages, are located in close proximity to neurons and play a role in gut motility in both health and disease [12,13].

**Figure 1:**
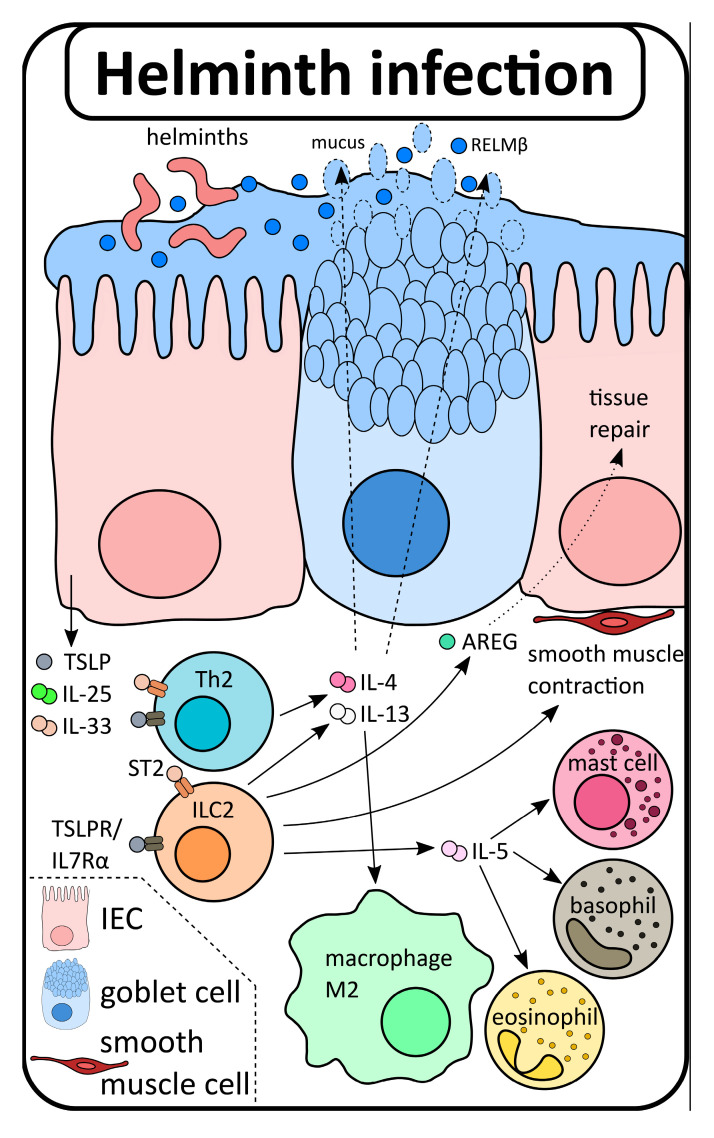
Type 2 immunity during helminth infection. Release of alarmins from IECs during helminth infection triggers the production of type 2 cytokines from Th2 and ILC2 cells, inducing smooth muscle contraction, tissue repair, immune cell recruitment, mucus and RELMβ production in order to expel the worms. AREG, amphiregulin; IEC, intestinal epithelial cell; IL, interleukin; ILC, innate lymphoid cell; RELMβ, resistin-like molecule-beta; Th, T helper cell; TSLP, thymic stromal lymphopoietin

Type 2 immunity most likely has evolved as a protective mechanism against helminthiasis (Figure 1). Upon parasitic infection, damaged intestinal epithelial cells release soluble factors known as alarmins, such as thymic stromal lymphopoietin (TSLP), IL-25, and IL-33. The best studied one of these is IL-33, an IL-1-like cytokine with a major impact on type 2 immune responses in the intestine, whose actions are mediated by the ST2 receptor complex [14]. As mentioned before, IL-25 is mainly produced by tuft cells, recently recognized as crucial initiators of the mucosal type 2 immunity during helminthic infection [15,16]. The importance of IL-33 and IL-25 during helminth infection is suggested by the higher susceptibility of mice lacking these alarmins [17]. Similarly, TSLP is constitutively expressed at barrier surfaces and upregulated after stimuli, such as the presence of helminths [18]. The result of the release of these alarmins is the activation and proliferation of Th2 cells as well as ILC2. Thereby, the crosstalk between Th2 and ILC2 is crucial for the optimal anti-helminthic response [19,20,21]. Upon activation, Th2 and ILC2 produce the classical type 2 cytokines IL-13 and, to a lesser extent, IL-4, which are the main drivers of response against helminths. In brief, these cytokines target tuft cells, goblet cells, and smooth muscle cells, inducing hyperplasia of the secretory compartment as well as the contraction of the latter [22]. At the same time, they have been shown to increase mucus production and the expression of the antiparasitic peptide resistin-like molecule-beta (RELMβ). Collectively, these effects constitute a protective strategy by supporting effective worm expulsion [22,23]. ILC2-derived IL-5 is another important mediator by inducing eosinophil recruitment, one of the central features of the response to helminthic infections [17]. Other effector cells such as mast cells, basophils, and alternatively activated macrophages are also recruited in response to epithelial damage [24]. In particular, by the release of soluble factors, these cells contribute to the amplification of type 2 immune responses, worm expulsion and, importantly, wound healing.

Their ability to modulate the immune response led to the hypothesis that helminths or parasitic-derived components could be broadly beneficial in some Th1/Th2 imbalance-related diseases, like IBD. For this reason, a potential therapeutic role of infections with different helminth species in different in vivo experiments and clinical trials were already tested recently. For example, *Trichinella spiralis* worms as well as helminth-derived molecules ameliorated mucosal damage in the setting of 2,4,6-trinitrobenzene sulphonic acid (TNBS) colitis [25,26,27,28,29,30]. In the same experimental model, metabolites derived from *Ancylostoma caninum* and *Schistosoma haematobium* similarly exhibited a protective effect in vivo [31,32]. Moreover, infection with *Syphacia obvelate* and *Metagonimus miyata* ameliorates murine dextran sulfate sodium (DSS) colitis [33,34]. In humans, so far two species have been used in clinical trials, namely, *Trichuris suis* and *Necator americanus* [35]. These trials have been conducted in both Ulcerative Colitis (UC) and Crohn’s disease (CD). The hypothesis underlying these clinical trials is that helminth infection will trigger a strong type 2 immune response that will oppose the excessive Th1 and Th17 response associated with CD (see below). At the same time, the chronic infection will generate a network of T helper cell (Treg) cells, which, in turn, will regulate both Th1 and Th2 responses in CD and UC, respectively [36]. Until now, promising results were obtained in these studies, and these therapies seem to be well tolerated. However, the use of living helminths remains controversial because of ethical concerns and practical issues [37].

## 3. The Role of Type 2 Immunity in IBD

IBD is represented by two clinically different conditions, namely Ulcerative Colitis (UC) and Crohn’s disease (CD), traditionally associated with a different cytokine profile: CD patients show a Th1-cytokine associated profile, whereas some type 2 cytokines are seemingly upregulated in UC. However, the classical concept of a Th1/Th2 imbalance has been challenged with the discovery of Th17 cells. These cells are characterized by the expression of IL-17, a cytokine strongly associated with both CD and UC [38]. Moreover, IL-4, one of the most important Th2 cytokines, exhibits lower levels in UC patients than in CD patients, an observation that further questions the definition of UC as a predominant type 2 mediated disease. Recent studies implicate the presence of a far more complex immune-regulating network in chronic intestinal inflammation exemplified by the presence of Th1, Th2, Th9, Th17, and Treg cells. Moreover, cell populations characterized in the lamina propria of IBD patients expressing Foxp3^+^ Treg and, simultaneously, IL-17 and RORγt expressing Th17 cells, have further increased the complexity of immune regulation in the gut [39,40].

Chronic inflammation and dysregulated immune responses are hallmarks of both UC and CD. In the case of UC patients, as stated above, an atypical type 2 cytokine pattern was described, with enhanced levels of IL-13, TGFβ, IL-5, or IL-33, and expression of GATA binding protein 3 (GATA3) in lamina propria T cells [40,41]. Yet, conflicting results regarding the levels of IL-13 in the inflamed mucosa of UC patients have been published, showing either an increased expression of IL-13 or the opposite result in other studies [42,43]. Regarding the role of IL-13 in experimental models of intestinal inflammation, IL-13 blockade ameliorates colitis induced by oxazolone, a murine model resembling some features of UC. A similar result was obtained after using a bifunctional IL-4/IL-13 antagonist in the same murine model. In further support of this observation, another study revealed an alteration in tight junction functionality and a direct apoptosis-initiating effect of IL-13 in epithelial cells. The pro-inflammatory function of this cytokine was also underlined by demonstrating an association between increased levels of IL-13 and enhanced intestinal permeability [44,45,46]. Moreover, Karmele et al. have shown a positive effect of anti-IL13Rα2 therapy in a different experimental model, namely chemically induced DSS colitis [47]. Finally, another study revealed that GATA3 transgenic mice exhibited more pronounced colitis induced by DSS, a phenotype associated with a higher intestinal production of IL-13 [48]. In light of all these observations in mice, one could conclude that IL-13 is a driving factor in the development of intestinal inflammation and that blockade of IL-13 could be a promising therapeutic approach. However, this hypothesis has suffered a setback as the result of two clinical trials have failed to demonstrate the efficacy of anti-IL-13 therapy (anrukinzumab and tralokinumab) in IBD patients, largely questioning a relevant role of IL-13 in driving IBD [49]. While there is currently no widely accepted explanation for this failure, one reason could be the overlapping actions mediated by IL-4 and IL-13. In fact, both cytokines require the same receptor subunit, IL-4Rα, which is part of a heterodimeric receptor complex of each cytokine. Another explanation could be the pleiotropic functions of these cytokines acting on different target cells in a temporally and spatially regulated manner. For example, while blocking IL-13 in some preclinical models prevents the development of a clinical disease, IL-13 and IL-4 might have additional important functions during recovery and resolution of inflammation in IBD.

Besides IL-13, the role of IL-4 seems to be controversial as well. In IBD, although IL-4 is undetectable at the mRNA level in the mucosa of both UC and CD, a significant increase of serum IL-4 was detected in the acute phase of pediatric UC, with no changes in CD patients. Moreover, supporting the role of IL-4 in the pathogenesis of IBD, IL-4 polymorphisms have been associated with predisposition to this disease [50,51]. In oxazolone-induced colitis, the administration of anti-IL-4 led to the amelioration of disease, suggesting a detrimental role of this cytokine in experimental intestinal inflammation. In opposition to this harmful effect of IL-4, Xiong et al. described that treatment with an IL-4 expression plasmid reduced disease severity and levels of pro-inflammatory cytokines in a TNBS colitis model [52]. These discrepancies could be attributable to the different mouse models used. Results from oxazolone colitis might create a rationale for anti-IL-4-therapy as a candidate in the treatment of UC. However, to date, no clinical trials using anti-IL4-therapy have been conducted in IBD. Little is known about the role of IL-5 in IBD, although augmented levels of this cytokine could promote eosinophil recruitment to inflamed tissue in UC [50].

The type 2 cytokines IL-4, IL-5, and IL-13 are also considered crucial mediators of food allergy and an increased type 2 immune response is a feature of this disorder [53]. Consequently, it is possible to hypothesize that the existence of food allergy creates an inflammatory milieu with a potential impact on IBD pathogenesis. Indeed, Li et al. showed that in mice, food allergy induces an IBD-like inflammation in the colon [54]. In further support of a link between food allergy and IBD, IgE has been associated with the pathogenesis of both disorders: Accordingly, patients with allergies have an altered response of IgE to innocuous antigens and, in IBD patients, eosinophilia has been suggested to be mediated by IgE [55]. Moreover, an increased level of food-specific IgGs in comparison with healthy subjects has been described in CD patients [56]. Importantly, at present, no clear functional data has been reported to causally link food allergies to the disease course of IBD.

IL-4 and IL-13 are also involved in intestinal permeability, as shown by the reduced intestinal resistance observed after the exogenous administration of these cytokines in mice [57]. In vitro, IL-13 has been demonstrated to regulate tight junction proteins and epithelial sodium channels [58,59]. Interestingly, there is a correlation between genes involved in intestinal permeability and IBD susceptibility. Moreover, increased paracellular permeability and altered tight junctions are hallmarks of IBD. Independently, the consequence of a leaky gut is the translocation of gut microbial components, which, in turn, triggers an inflammatory response. In fact, systemic endotoxemia is a common observation in IBD (28–88% in UC and 48–94% in CD during clinical relapse) [60]. These data collectively suggest that increased type 2 cytokine responses contribute to the pathophysiology IBD by compromising epithelial barrier functions. However, if such altered permeability is the cause of the exacerbated inflammatory response or a consequence of the established inflammation is still poorly understood [61].

## 4. Type 2 Associated Immune Cells in IBD

**Figure 2:**
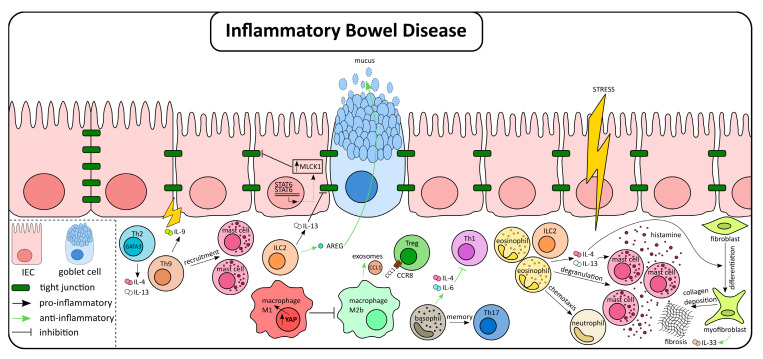
Schematic representation of IECs and immune cell crosstalk, mediated by type 2 cytokines, during inflammatory bowel disease. Dual effects of type 2 cytokines and effector cells during intestinal inflammation. CCL1, C-C chemokine ligand 1; CCR8, C-C chemokine receptor type 8; GATA 3, GATA binding protein 3; MLCK1, myosin light chain kinase 1; STAT, Signal transducer and activator of transcription; Treg, T helper cell; YAP, Yes-associated protein.

Type 2 effector cells are present in chronic intestinal inflammatory diseases, including IBD (Figure 2). This population includes cell types belonging to both the adaptive and the innate immune system. The former includes Th2 cells, which are characterized by the expression of the transcription factor GATA3. The contribution of GATA3 in colitis has been investigated in several publications with data from mice and humans, and especially from UC patients. Accordingly, in pediatric patients, while no changes were detected in GATA3 expression in CD, an increase was described in the case of UC [51]. A similar observation was made when samples of adult UC and CD patients were analyzed, demonstrating an enhanced expression of GATA3 in CD4^+^ cells exclusively in UC. Studies performed in mouse models have further evidenced the enrichment of the GATA3^+^CD4^+^ T cell population in intestinal inflammation. Using the oxazolone-induced colitis model, Popp et al. detected an augmented percentage of GATA3^+^CD4^+^ cells in the lamina propria of treated animals. Moreover, they confirmed that GATA3 has a pathogenic role in this context since the conditional deficiency of GATA3 in T cells protected mice from oxazolone colitis [62]. In order to determine the role of T helper cells in a different model, Okamura et al. induced DSS-colitis in T-bet, GATA-3, and RORγt transgenic mice. Again, they found that only GATA3 overexpression led to more severe colitis with enhanced levels of IL-13, which was causatively involved in the development of colitis [48]. Th9 cells, a recently discovered additional player in adaptive immune responses, seems to be also involved in the pathogenesis of IBD. In UC patients, an augmented number of Th9 cells has been reported, as well as a positive correlation between Th9-derived IL-9 and disease progression in IBD. This observation might be explained by the ability of this cytokine to alter tight junction protein expression and consequently, gut permeability. The latter could be the effect of a direct or indirect mechanism, that is, Th9 can act amplifying type 2 immunity, e.g., via recruitment of other cell types, such as mast cells. This cell type has a deleterious impact on gut permeability through the release of histamine and PGE_2_ [57,63,64,65]. However, more research is needed in order to shed light on the precise role of Th9 cells in IBD.

Similar to Th2 cells, ILC2s are an important source of type 2 cytokines, defining this innate immune cell population as a potential player in IBD. In contrast to Th2 cells, enhanced numbers of ILC2 could be detected in both UC and CD. Of note, this was true only in established IBD, whereas in newly diagnosed patients, the expansion of ILC2 was solely detectable in UC. Interestingly, two studies described the presence of IFNγ-secreting ILC2, an observation that demonstrates the remarkable plasticity of ILC populations [66,67]. Other reports have focused on the role of ILC2 in experimental colitis, with an inconclusive picture so far. In the DSS model, ILC2 was described as a major source of IL-13. In this study, IL-13 contributed to gut permeability and intestinal inflammation via activation of STAT6 and subsequent dysregulation of tight junctions through upregulation of myosin light chain kinase 1 (MLCK1) [68]. However, the conclusion of a pro-inflammatory role of ILC2 is in contradiction with another study, in which the role of IL-33 in the pathogenesis of IBD was analyzed. In this report, the authors showed that IL-33 ameliorates DSS-induced colitis and concluded that the protective mechanism involved stimulation of ILC2 and their subsequent production of amphiregulin (AREG) [69]. In further support of this interpretation, ILC2-derived AREG, a ligand of the epidermal growth factor receptor, improved intestinal disease and promoted mucin production. Altogether, the role of ILC2 in IBD is still poorly understood and further investigations are needed to better define their functional contribution to the different stages of inflammation.

Resident macrophages are another important contributor to type 2 immunity in the gut. Traditionally, macrophages are divided into two different subtypes: M1-like and M2-like, with pro-inflammatory and anti-inflammatory features, respectively [70]. Several studies established that M1-like macrophages expand and accumulate in the colon during colitis, although substantial numbers of M2-like macrophages are also present. Whereas M1 acts by rather promoting inflammation, an inflammation-attenuating effect has been described for M2. In fact, adoptive transfer of M2 macrophages in mice with experimental colitis induced by DNBS and DSS leads to a milder disease [71,72,73]. This seems to be in line with the efficacy of some PPARγ ligands in experimental models, which are known to promote M2 polarization [74]. Macrophage polarization is regulated by different transcription factors, such as Yes-associated protein (YAP). Recently, a publication has revealed the role of YAP in macrophages fate and the outcome of experimental colitis. YAP acts by promoting M1 polarization together with an inhibition of M2 polarization, a setting that aggravates colitis [75]. In a different study, loss of Fibrinogen-like protein 2 (Fgl2) was associated with M1 polarization and increased susceptibility to DSS treatment. Similarly, peritoneal macrophages from Fgl2^-/-^ mice exhibited a reduced polarization towards M2 when stimulated [76]. Interestingly, these studies pointed to different mechanisms of M2-like macrophages to provide protection from intestinal inflammation. For instance, M2-like macrophages were described to alleviate colitis through the release of exosomes. Colon exosomes from a specific M2 subpopulation, M2b, contained the chemokine C-C chemokine ligand 1 (CCL1). Authors described a protective role of these M2b-derived exosomes mediated by the interaction of CCL1 and CCR8, which lead to the induction of IL-4 expression and the increase in the number of Treg cells [77]. Furthermore, M2-like macrophages are crucial during the repair phase after intestinal inflammation by activation of the WNT signaling pathway in IECs [78].

Just a few studies examined the role of basophils in IBD. However, an analysis of the mucosa of IBD patients revealed an accumulation of basophils. Interestingly, basophils have been reported to contribute to the exacerbation of inflammation and Th17 cell-dependent immune responses, both in in vivo and in vitro studies [79,80]. However, evidence from other animal studies revealed a rather protective role of basophils. This was described in a model of T-cell transfer colitis, where the lack of basophils promoted pro-inflammatory Th1 responses and colitis exacerbation. According to this report, basophils are protective, because basophil-derived IL-4 and IL-6 promoted type 2 immunity and suppressed the production of Th1 cytokines [81]. This effect of basophils on amplification of Th2 responses was also observed in the skin, where inflammatory monocytes switched to an anti-inflammatory phenotype in response to the release of IL-4 from basophils, dampening the allergic response [82].

An extensive review focusing on the function and role of eosinophils in the gut and colorectal disease has recently been published [83]. In brief, it has been described that eosinophils produce cytokines linked to both Th1 and Th2 immunity. However, in the intestine, eosinophils have been associated with the production of IL-4 and IL-13. Similar to basophils, IBD patients exhibit a pronounced infiltration of eosinophils in the mucosa, especially in UC patients, not only in active disease but also during remission. The presence of eosinophils has even led to suggest eosinophil-derived markers, such as eosinophil-derived neurotoxin (EDN) and eosinophil cationic protein (ECP) as fecal markers in IBD, complementary to calprotectin [84,85]. Mechanistically, it is considered that eosinophils are effector cells that contribute to barrier dysfunction through the release of pro-inflammatory cytokines and cytotoxic factors after degranulation. At the same time, they can amplify inflammation by the promotion of mast cell degranulation and neutrophil chemotaxis [83]. In support of this, UC patients exhibit impaired barrier function even during remission. Along the same line, eosinophil numbers are correlated with paracellular permeability, suggesting the involvement of this cell population in regulating mucosal permeability [86]. However, there are conflicting results regarding the association of eosinophil numbers and disease severity in humans. This is revealed by a recent study reporting that patients with eosinophil-predominant inflammation have a lower risk of disease flares and hospitalization than those with a neutrophil-predominant inflammation [87]. It is worth noting that an increased number of eosinophils is a hallmark not only of IBD but also of a different disease, known as eosinophilic colitis (EC) [88]. This disorder is erroneously diagnosed as IBD in around 25% of the patients with EC. The reason for this misdiagnosis is the presence of common clinical manifestations and pathogenesis.

Few studies have analyzed the role of mast cells (MC) in IBD, although the mucosal accumulation of MC is a common observation in IBD. These studies point to a deleterious impact of MC on IBD development. Hence, in mouse models of acute colitis, either inactivation or depletion of MC leads to an ameliorated inflammation [89]. In the inflamed intestine, impaired intestinal barrier function and dysbiosis induce MC responses, which lead to the maintenance of an inflammatory milieu and an increase in gut permeability. Proteases and pro-inflammatory cytokines are responsible for these effects while neuromodulators such as histamine, tryptase, or substance P contribute to pain and altered peristalsis in IBD patients. Stress is an event that can connect IBD and MC. Stress triggers eosinophils and MC, which, in turn, can promote intestinal permeability and stress-related flares in IBD [90,91]. Altogether, these studies point towards a deleterious impact of MC on IBD development. MC numbers are also increased in a different intestinal pathology closely linked to stress, such as the irritable bowel syndrome (IBS), in which MC are generally recognized as mediators of the main IBS symptoms [92,93].

## 5. Type 2 Immunity Responses in the Context of Colorectal Cancer

Colorectal cancer (CRC) is the third most common cancer and the fourth cause of cancer death worldwide [94]. In a meta-analysis published in 2013, authors identified several colorectal cancer risk factors. Lack of physical activity, smoking, and poor diet were associated with a moderately increased risk, while the highest CRC risk was found in patients with a family history of CRC or patients with IBD [95]. Supporting this view, a recent publication identified extensive UC and perforating CD as significant risk factors for any malignancy [96]. Colitis-associated cancer (CAC) accounts for 1-2% of all CRCs. In spite of the different cytokine profiles in UC and CD, as described above, both conditions have a comparable cancer risk, a circumstance that reveals the complexity of the relationship between immune response and cancer development [97,98,99]. The fact that obesity, which is a driver of continue and low-grade inflammation in the body was identified as a risk factor too, further underlines the connection between inflammation and cancer.

Tumors are characterized by a heterogeneous collection of cells. In addition to cancer cells, tumors include a complex network of molecules known as the extracellular matrix and several types of cells involved in the development and progression of CRC. Together, these components form the tumor stroma. Fibroblasts, endothelial, and immune cells are the components of the cellular fraction of the stroma and both pro-tumoral and anti-tumoral effects have been attributed to different stromal cell populations. [100]. Antitumor immunity and tumor cell killing are usually associated with a type 1 immune response. Anti-tumoral functions are mainly executed by NK cells, recruited monocytes, and CD8^+^ cytotoxic T cells. NK cells trigger the killing of cancer cells that lack MHC-I molecules. Macrophages and dendritic cells present tumor-associated antigens to the T cells, which, in turn, can directly lyse cancer cells. On the contrary, there are stromal cells characterized by a type 2 immune profile. These cell types have attracted increasing attention during the last years due to the repeated observation that a predominant type 2 immune response in inflammatory conditions is associated with colonic tumor development in mice, although this is not applicable to all immune cell populations.

In fact, the involvement of type 2 immunity in the development of cancer is clearly reflected by the association between IL-4, IL-13, and IL-4Rα gene polymorphisms and CRC [101]. During tumor development, an increased expression of IL-4R, IL-4 as well as IL-13 and its high affinity receptor IL-13Rα2 are detected in some epithelial cancers, including CRC. Several lines of evidence support a pro-tumor effect of IL-4 and IL-13 in vivo. In epithelial cells, IL-4 acts via STAT6, a pathway that is correlated with CRC. In agreement with the pro-tumorigenic role of this type 2 immune pathway in the Adenomatous polyposis coli (APC)^min/+^ model of intestinal tumorigenesis, disruption of STAT6 led to reduced formation of polyps [102,103,104,105]. The association between chronic inflammation and CRC has been explored using azoxymethane (AOM) and DSS as a model of CAC. In accordance with the data from APC^min/+^ mice, IL-4 has a positive impact on tumor progression in CAC, as shown by the reduced growth of tumors after AOM/DSS treatment in the absence of IL-4 [105]. Another chronic inflammation scenario is obesity. Induction of cancer development using AOM in obese and control mice showed a higher number of colorectal tumors in the former. This observation seemed to be connected to type 2 immunity since obese mice had significantly higher levels of IL-13 in the serum and an augmented expression of IL-13 receptor in colon tissue, which led authors to propose the involvement of this cytokine in the development of obesity-related CRC [106].

Additional pieces of evidence for the involvement of type 2 immunity in CRC were obtained using CRC cell lines and xenograft tumor models. Accordingly, it has been shown that IL-13 induces epithelial-mesenchymal transition (EMT) via STAT6 in colon cancer cell lines, a process that was reversed by propofol, due to the ability of this anesthetic agent to suppress the expression of STAT6 [107,108]. In addition, IL-13 could mediate a prometastatic effect in CRC through IL-13Rα2 [103]. In support of this mechanism, inhibition of ligand–receptor binding using a synthetic peptide or blocking the scaffold protein FAM 120A, necessary for IL13Rα2-mediated signaling, has demonstrated antimetastatic activity in vitro and in vivo as well as an augmented survival to CRC in vivo [109,110]. In in vitro experiments, treatment with anti-IL-4 reduces the expression of CD133 in the Caco2 cell line, a marker of cells with stemness properties, such as self-renewal and increased proliferation [111]. Moreover, both IL-4 and IL-13 contribute to the increased production of reactive reactive oxygen species (ROS) in CRC, which, in turn, contributes to the development of inflammation-mediated malignancies [112,113]. The NADPH oxidase family is one of the most relevant sources of ROS during cancer development NADPH oxidase 1 (NOX1) and Dual oxidase 2 (DUOX2) are examples of upregulated enzymes in tumor tissue. ROS derived from these enzymes contributes to DNA damage and neoplasia. In vitro experiments using HT-29 cells revealed the ability of IL-4 and IL-13 to induce the upregulation of NOX1, whereas DUOX2 expression was increased after IL-4 treatment in the T84 cell line. This mechanism helps to establish an oxidant milieu, a driver of inflammation-dependent cell proliferation and tumor progression.

Although controversial, evidence points out an important role of the alarmin IL-33 in intestinal tumorigenesis [114]. IL-33 is elevated in CRC, both in tissue and serum samples, and the IL-33/ST2L axis shapes immune cell populations in the tumor, promoting the accumulation or depletion of immunosuppressive and anti-tumorigenic cells, respectively [115,116]. Moreover, the IL-33/ST2L axis is critical for Treg expansion and, therefore, it could indirectly promote tumor suppression. Accordingly, IL-33 administration to CT-26 (murine colon carcinoma cell line) tumor-bearing mice promotes an increase in the ST2^+^ Treg population, which, through the production of Th2 cytokines, promotes cancer development [117]. By contrast, Long et al. recently described that IL-33 suppresses tumor growth in a xenograft cancer model in Rag1^-/-^ mice [118]. Interestingly, in the same study, the anti-tumor effect of IL-33 was potentiated after the depletion of ILC2, indicating a tumorigenic effect of this cell population in the presence of IL-33. The authors went on to demonstrate that ILC2 expanded in the presence of IL-33 inhibited the anti-tumor effect of NK cells. In further agreement with the tumor-promoting effect of type 2 immunity in the gut, IL-33 transgenic mice inoculated with MC38 cells exhibited an increased cell proliferation, an effect driven via cyclooxygenase (COX)/PGE2 [119]. On the contrary, IL-33 suppresses the growth of HCT-116 colorectal tumor cells [120]. Collectively, these observations reflect the complex role of IL-33 in CRC, with different results in different contexts. Further studies will be necessary to better understand these observations and to reveal the contribution of this cytokine to the different stages of colonic tumor development and its specific functions on tumor cells and the cells of the tumor stroma.

## 6. Type 2 Immune Cells in CRC

**Figure 3:**
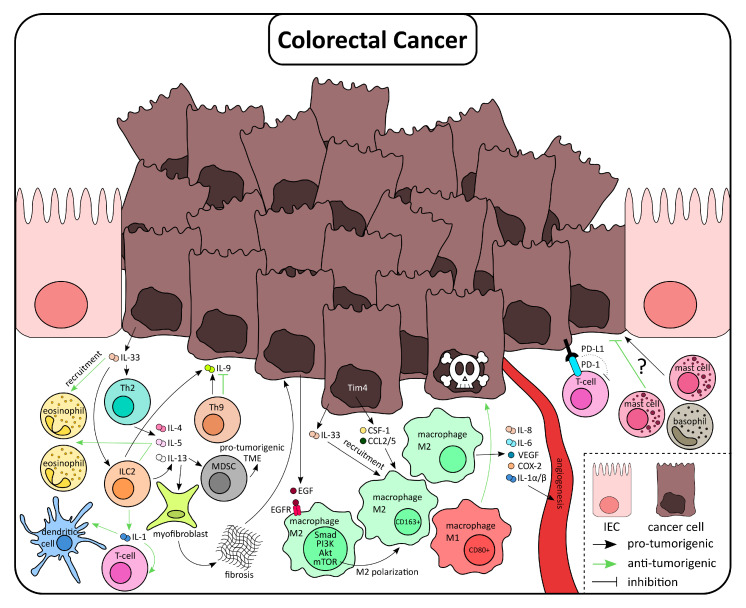
Schematic representation of IECs and immune cell crosstalk, mediated by type 2 cytokines, during colorectal cancer. Type 2 immune responses exhibit pro- and anti-tumorigenic effects during colorectal cancer development. Akt, protein kinase D; COX-2, cyclooxygenase 2; CSF-1, colony-stimulating factor 1; EGF, epidermal growth factor; MDSC, myeloid-derived suppressor cell; mTOR, mammalian target of rapamycin; PD-1, programmed cell death protein 1; PD-L1, programmed death ligand 1; PI3K, phosphatidylinositol 3-kinase; TIM4, T-cell immunoglobulin and mucin domain containing 4; TME, tumor microenvironment; VEGF, vascular endothelial growth factor.

As mentioned before, stromal cells include cell types that belong to type 2 immunity, including components of the innate and adaptive immune response (Figure 3). In the latter, it has been shown that predominance of Th2 cells in CRC is associated with a worse prognosis, mainly due to the release of IL-4 [121]. In support of this, in a recent publication, a connection between Th1/Th2 imbalance and the poorer prognosis was described [122]. The deleterious effect of Th2 is further suggested by the greater number of tumors in a mouse model of CAC in IFNγ^-/-^ mice [123]. Since these mice have augmented levels of IL-4 and IL-5, the authors pointed to the contribution of a dominant Th2 response to the observed phenotype. Th9 cells, a cell type also strongly related to type 2 immunity, seems to have a more controversial role in CRC. In two clinical trials in China, it was described that patients with CRC had lower expression of IL-9 in plasma and CRC tumor tissue, and the expression level was correlated with the tumor stage: the more advanced the tumor stage, the lower the IL-9 serum level. The opposite finding, that is, higher levels of serum IL-9 in CRC patients, was described in a recent study from Poland [124]. Again, debatable results have been published regarding the anti- or pro-tumor effect of Th9 cells in experimental models of CRC and in vitro experiments. While some studies claimed that IL-9 inhibits tumor growth both in vivo and in vitro, others have found that IL-9 increases proliferation in some tumor cell lines and its absence contributes to the early rejection of transplanted tumor cells in vivo [124].

Recently, ILC2 cells have been introduced in tumor biology. The functions of ILC2 and Th2 during type 2 immune responses are similar and, as Th2, ILC2 produce both IL-4 and IL-13. The role of ILC2 in CRC has been extensively reviewed by Atreya et al. only recently [114]. The involvement of ILC2 in CRC is suggested by the increased frequency of ILC2 in patients with colonic inflammation, a condition linked to a higher risk of this inflammation-driven-CRC [114]. In support of a pro-tumorigenic role, high levels of the ILC2-derived alarmin IL-33 and IL-4 were associated with poor prognosis [125]. Moreover, ILC2-derived IL-13 activates myeloid-derived suppressor cells (MDSC), which, in turn, promote a tumorigenic microenvironment [107,126]. However, some anti-tumor roles have also been claimed. For example, ILC2 releases IL-5, a mechanism by which ILC2 induce eosinophil expansion IL-5 expression was associated with better prognosis. Moreover, through the secretion of IL-13, ILC2 have been shown to promote DC migration and cytotoxic T cell activation, which might support anti-tumor immunity [125]. With divergent results, it is currently not possible to conclude upon the role of ILC2 in cancer biology. Further well-designed studies will be needed to provide more information about the functional role.

A major immune cell population infiltrating tumor tissue in CRC is macrophages. Tumor associated-macrophages (TAM) are largely M2-like macrophages. The overall survival is reduced in patients with elevated M2-like macrophage numbers, an observation that underlines the pro-tumorigenic role of this macrophage subtype [127]. A general explanation for these observations is that classically activated macrophages exhibit anti-tumor function by direct killing of tumor cells, whereas, in the tumor microenvironment, the characteristic wound healing response of M2-like alternative activated macrophages promotes tumor cell growth [128]. M2 cell recruitment and macrophage polarization are promoted by the tumor microenvironment [129], for example, through the release of IL-33. In the context of an APC gene mutation, IL-33 exhibits a deleterious effect, partly mediated by the induction of type 2 cytokines and M2-like macrophage polarization [130]. Other mechanisms promoting macrophage recruitment include the increased expression of PIPKI*γ* in cancer cells, an enzyme involved in regulating cell migration [131]. PIPKI*γ* mediates the production of the monocyte-attracting chemokine CCL2. In further support for the important role of macrophages, an inverse correlation between expression of PIPKI*γ* and overall survival has been described in various types of cancer, including CRC [132]. Furthermore, tumor cells release colony-stimulating factor 1 (CSF-1), CCL2, and CCL5, promoting the recruitment of macrophages. The expression of these chemokines was dependent on the induction of the transcription factor Tim-4. [133]. This seems to support tumor growth, as high levels of Tim-4 predict poor prognosis in CRC patients. Recently, succinate was identified as a cancer progression factor released by tumor cells [134]. Through a mechanism involving the succinate receptor, SUCNR1, and the phosphatidylinositol 3-kinase (PI3K)/HIF-1α pathway, succinate induces the recruitment of macrophages and TAM polarization to M2-like phenotype. A different pathway involved in M2 polarization is Smad-PI3K-Akt-mammalian target of rapamycin (mTOR). According to Lian et al., human cancer cell lines can induce the M2 polarization of THP-1 cells via a mechanism involving epidermal growth factor (EGF)/EGFR signaling [135]. EGF/EGFR interaction in macrophages induced the activation of Smad-PI3K-Akt-mTOR and subsequently M2 polarization. Additional components of the stroma can induce M2 polarization. This is, for example, the case for cancer-associated fibroblasts (CAF). Accordingly, it has been demonstrated that M2-like macrophages and CAF synergistically suppress NK cell activity [136].

In spite of previous observations, the specific role of TAM is complex and controversially discussed due to conflicting results regarding its positive or negative impact on patient survival, although increasing evidence suggests that TAM have a pro-tumoral activity [129,137,138,139]. In agreement with the aforementioned tumorigenic role, blocking M2-like polarization in CRC seems to be effective. This has been demonstrated by Fenretinide treatment, a synthetic derivative of all-trans retinoic acid, which inhibits IL-4/IL-13-mediated STAT6 phosphorylation in vitro. A marked decrease of tumorigenesis and M2-like cell populations was observed after the administration of Fenretinide in APC^min/+^ mice [140]. Numerous mechanisms have been proposed to explain the pro-tumoral activity of TAM, namely, an impact on angiogenesis, immunosuppression, tumor growth, and induction of metastasis. For instance, macrophages release the proangiogenic cytokines IL-8, IL-6, vascular endothelial growth factor (VEGF), IL-1α, and IL-1β via p38/MAPKAP Kinase 2 [141], suggesting that intratumor macrophages are involved in intratumor vessel formation. The production of TGF-β by recruited macrophages assists tumor proliferation and invasion via EMT and VEGFR expression [142,143]. Moreover, TAM may induce CRC development due to immunosuppressive effects mediated by Programmed cell death *protein* 1 (PD-1). This receptor can be found on a wide range of cell types but is typically expressed on T cells where it mediates the suppression of cytotoxic activity. CCL5, one of the TAM-released chemokines, promotes the stabilization of PD-L1, the ligand of PD-1, via CSN5 expression. In support of these findings, human data showed that high CSN5 expression is associated with poor survival [144]. In a tumor, CD11b^+^CX3C1^+^ macrophage subset has been shown to induce CD4^+^ Foxp3^+^ cells, and its depletion leads to reduced tumor growth [145]. This immunosuppressive effect of TAM is further influenced by the hypoxic condition of the tumor microenvironment. In this context, FoxO1, a critical transcription factor for macrophage polarization, is downregulated. Under this condition, M2 polarization is induced and the expression of MHC-II molecules is reduced [146]. Regarding the role of macrophages in metastasis, it has been recently reported that incomplete radiofrequency ablation of tumors leads to the development of earlier metastases and reduced efficacy of PD-1 therapy. The authors of this study hypothesized that tumor-derived-CCL2 is crucial for the recruitment of macrophages, which exert immunosuppressive effects [147].

Lately, several studies have shed light on macrophage distribution inside the tumor. They collectively concluded that both M1-like and M2-like populations are not evenly distributed in the tumor tissue. In one of these studies, the authors used CD80 and CD163 as markers of M1-like or M2-like macrophages, respectively, together with CD68 [138]. The total number of CD68^+^ cells was increased in tumor tissue when compared to the adjacent normal mucosa. While the authors found a high number of macrophages at the invasive front, CD80 was mainly expressed in the adjacent normal mucosa and only scarcely in the intratumor regions. Only 40% of the macrophage population at the invasive front were positive for CD163^+^, indicative of an M2-like phenotype, suggesting that more than half of the macrophages at this location do not express any of these phenotypical markers. The authors of this study hypothesized that tumor macrophages exhibit a high heterogeneity. The same study revealed that CD163^+^ cells are more prevalent in stage II cancer tissue, whereas CD80^+^ macrophages are predominant in less invasive T1 tumors. Again, this underlines a potential link between M2-like macrophages and a poor clinical prognosis. A higher ratio of CD163^+^/CD68^+^ at the invasive front in comparison with the tumor center was also described elsewhere [148]. Interestingly, in this study, the ratio of CD163^+^/CD68^+^ at the invasive front seemed to be correlated with the expression of EMT markers as well as with clinicopathological parameters, proposing the use of this ratio as a prognostic marker of CRC treatment because of its association with poor prognosis. A similar result was obtained by Wei et al., who described that increased numbers of CD163^+^ cells at the invasive front were correlated with EMT, mesenchymal circulating tumor cell (CTC) ratio, and poor prognosis of CRC [149]. However, one important limitation of these studies is the analysis of a sole marker to identify the two macrophage subpopulations. Moreover, there is a discrepancy between in vitro and in vivo M1/M2 polarization that could explain why some makers, well-characterized in in vitro experiments, fail to translate in macrophages in vivo [150]. Hence, a better characterization of phenotypic macrophage markers is required to better characterize the imbalance between M1 and M2-like macrophages at the invasive tumor front.

In further support of the tumor regulating role of type 2 immunity, eosinophilic granulocytes are increased in tumor tissue and peripheral blood, and this increase has been associated with better prognosis. In CRC, it seems to be a link between an increased presence of eosinophils in tumor tissue or blood, and a better prognosis [83,151,152]. In the APC^min/+^ mouse model of CRC, an accumulation of eosinophils was again observed. Tumor-associated eosinophils exhibited a prolonged survival, a characteristic that was dependent on the TME but independent of IL-5, an important effector in eosinophils recruitment. In this study, eosinophils elicited a potent anti-tumor response that was unconnected with CD8^+^ cells [152]. The existence of a negative correlation between allergy and CRC risk and mortality has been described [153,154,155], however, specifically in the case of food allergy, there is not enough evidence to support this link with CRC [156]. The presence of eosinophils in the blood is a hallmark of allergic diseases and a negative correlation was described between blood eosinophil counts and CRC risk [157]. Moreover, the presence of eosinophils inside the tumor mass is negatively correlated with the presence of cancer cell deposits [158]. Andreone et al. described a direct anti-tumor effect of IL-33 activated-eosinophils on different tumor cell lines, MC38 colon carcinoma cells among others [159]. Interestingly, similarly to eosinophils, high levels of basophils and a better prognosis in CRC has led to the hypothesis that basophils could exert anti-tumor actions [160,161]. Given the fact that other type 2-related cells seem to have a positive effect on cancer development, it is possible that the negative correlation between allergy and CRC is attributable to eosinophils and basophils to some extent.

Mast cells (MC) are recruited early during tumorigenesis and they are mainly located around vessels. [162]. A number of human studies described conflicting results regarding the prognostic value of MC presence in the tumor. For example, on the one hand, a high number of MC in the tumor has been correlated with longer overall survival in CRC [163]. Similarly, using CD117 and mast cell tryptase (MCT) as MC markers, Giuşcă et al. revealed a positive correlation between peritumoral CD117^+^/MCT^+^ MC and survival of patients with liver metastasis [164]. On the contrary, other studies have demonstrated the opposite result, with high levels of MC associated with cancer progression. For instance, advanced stages of CRC and nodal dissemination were associated with a higher level of intratumor MCT^+^ MCs [165,166,167]. Moreover, in a meta-analysis published in 2018, the presence of MC infiltration, defined as trypsin+ cells, was associated with a decrease of overall survival and disease-free survival in several solid tumors, including CRC [168]. In support of the above findings, Mao et al. identified MC density as a prognostic factor negatively correlated with overall survival and reduced immune activation [169]. Recently, a MCs signature, useful as a proxy of MCs infiltration, has been proposed. These indices are higher in tumor tissue, reflecting an enhanced presence of MCs, and can predict clinical outcomes in colon cancer among others [170].

Considering the conflicting reports of MC and their value for CRC prognosis, it is not surprising that the effect of MC in cancer development is equally open to discussion. Although increasing evidence points to a tumorigenic activity, some publications describe anti-tumorigenic functions of MC, and the outcome seems to depend on the type of cancer and the stage of tumor development. MC has pro-inflammatory functions and, after activation, degranulate, releasing histamine, chemokines, and cytokines, and recruit other immune cells to exert anti-tumorigenic actions [162]. For example, MC express a high level of prostaglandin D and release of PGD_2_, which has been shown to protect from both colitis and colitis-associated colon cancer. In this context, MC-derived PGD_2_ suppressed the expression of TNF and MCP-1 in the inflamed tissue, a mechanism that was associated with the development of colon cancer [162,171]. Another relevant molecule in MC is histamine, one of the main mediators of MC activity. Histamine is produced by the enzyme histidine decarboxylase (HDC), which is primarily found in immature myeloid cells. The involvement of histamine in allergy and cancer has been studied in several publications. Histamine, after OVA immunization, induces a Th2 response in T cells. However, in HDC^−/−^ mice, OVA treatment leads to the increase of IL-17-producing MC. This MC population induces the recruitment of MDSCs and, in consequence, the immunosuppression of cytotoxic T cells and carcinogenesis in the AOM/DSS model of CRC. Authors of this study concluded that histamine acts by repressing IL-17 expressing MC and, as a result, reduces cancer risk in the context of food allergy [172]. In contrast to the above studies, cumulative evidence suggests a pro-tumorigenic activity of MC and histamine in CRC. This is highlighted by the possibility of the usage of antihistamine in cancer treatment, which has been reviewed elsewhere [173]. Recently, aside from histamine, a number of additional different tumor-promoting mechanism of MC has been proposed. Thus, within the tumor, MC release a number of angiogenic factors, such as IL-8, VEGF, FGF-2, NGF, TGF-β or tryptase [167]. MCs can also induce the expression of VEGF in cancer cell lines as well as the proliferation and migration in vitro, an effect mediated by the activation of MAPK/Rho-GTPase/STAT signaling pathways [166]. The release of IL-10, histamine, or adenosine from MC has been associated with an immunosuppressive pro-tumorigenic effect in CRC, whereas MMP9 release by MC was shown to promote metastasis. In CAC, it has been demonstrated that MC have increased levels of mMCP-1, a chemokine that induces the infiltration of CD11b^+^ Gr1^+^ cells, which contribute to tumor cell proliferation and inhibition of T cell activation, ultimately promoting cancer development [174]. Similarly, Danelli et al. demonstrated that MC possess pro-tumorigenic properties by recruiting MDSC cells and by potentiated their suppressive properties. In this study, it was demonstrated that this MC-mediated immune suppression depends on the collaboration with CD4+ cells [175]. Moreover, in the studies mentioned before, MC affected tumor development recruiting immune cells. However, recent studies demonstrated that MC can affect carcinogenesis by direct actions on cancer cells. In 2D and 3D coculture models, cancer cell lines release a number of mediators, such as CCL15 or SCF and thereby recruit MC. In turn, MC directly promoted tumor cell proliferation [176]. Like stem cells, MC also express beta-catenin, an important pro-proliferative molecule that is activated in response to Wnt ligands secreted by colonic tumor cells [177]. Strikingly, the activation of beta-catenin in MCs was shown to induce the formation of colonic polyps. The authors of this study went further and identified that these polyps showed a strong infiltration of MCs and a typical Th2 microenvironment, which, in turn, fuel further expansion of MCs. In the AOM/DSS model, experimental MC deficiency led to a higher IEC proliferation and the development of increased numbers of preneoplastic intestinal polyps. A deeper analysis of the neoplastic areas showed smaller and more differentiated tumors in those MCs-deficient mice. In conclusion, during persistent inflammation, the lack of MC could contribute to increased transformation but also to a reduction of the grade of the developing tumors [178]. To date, the precise role of MC in CRC is not fully understood. As shown above, under some experimental conditions, MC promote tumor development, whereas other studies reported anti-tumorigenic activities. Some of these discrepancies might be attributable to the experimental conditions used or to the specific cancer model. For example, MC might play a different role in colitis-dependent models than in colitis-independent models. Moreover, MC and their mediators likely have pleiotropic functions, some of which might be more or less important under different experimental conditions. Collectively it is clear that further studies will be needed to understand the role of a type 2 dominated tumor microenvironment.

## 7. Conclusions

In the last years, our knowledge of the roles of type 2 immunity in cancer and immune-related diseases has increased dramatically. As a result of extensive research, the involvement of type 2 immunity in gut homeostasis as well as during pathogenic conditions has been revealed. The natural type 2 immune response is directed against helminthic infections, which is host protective and leads to the killing of the parasite and/or to the expulsion of the worms. Moreover, type 2 immunity has been recently associated with wound healing and the resolution of inflammation, such as seen in patients with IBD. While chronic inflammation is triggered by Th1 and Th17 immune responses, the immune modulation induced by parasitic infection gives rise to the question of whether helminth infection or parasitic-derived components could be used in the therapy of diseases characterized by a Th1/Th2/Th17 imbalance. In this context, several clinical trials have been conducted in IBD with promising results, although the use of this therapy remains controversial [37]. However, more research is needed to understand if ongoing Th1 and/or Th17-dominated inflammation can be reprogrammed by promoting type 2 immunity. In the case of CRC, a rather pro-tumorigenic role has been associated with type 2 immunity, a circumstance that raises further concerns about the risks of therapy promoting type 2 immunity in patients with chronic inflammation and potentially undiscovered neoplastic lesions. Thus, in summary, the role of type 2 effector cells in CRC is poorly understood. Open questions also remain regarding the initiation of type 2 immunity in the gut and the cell types involved. As mentioned above, Th2 cells and ILC2 produce type 2 cytokines. Their activation, however, requires cytokines such as IL-33 or IL-4, leaving the question of the initial source of these cytokines. In a recent report, resident fibroblasts were shown to be producers of IL-33 during inflammatory conditions [165]. Whether fibroblast-derived IL-33 is a major player in the skewing of intestinal immunity to type-2 dominated responses remains to be investigated. A better understanding of these mechanisms is crucial and may help to develop future strategies for the treatment of both IBD and CRC.
